# Supplementary material for: Metabolic Profiling in Tuberous Roots of Ranunculus asiaticus L. as Influenced by Vernalization Procedure
Source: Plants (Basel). 2023 Sep 13;12(18):3255. doi: 10.3390/plants12183255 (PMC10537181; doi:10.3390/plants12183255)

Supplemental Table S1. Soluble proteins (in mg g^{-1} DW) and free amino acids (in μmol g^{-1} DW) in plants of *Ranunculus asiaticus* L. hybrids MDR and MBO, obtained by three vernalization procedures of tuberous roots, only rehydration (Control, C), rehydration plus vernalization for 2 weeks (V2), rehydration plus vernalization for 4 weeks (V4), throughout the growing cycle, in relation to the different plant stages (pre-planting, vegetative phase and flowering). Amino acids (AA), γ-aminobutyric acid (GABA), monoethanolamine (MEA), branched-chain amino acids (BCAAs). Values represent means of three replicates. Different lowercase or capital letters within each row, for specific vernalization procedure, indicate significant differences ($p \leq 0.05$). ns, *, ** and ***; indicate non-significant or significant difference at $p \leq 0.05$, $p \leq 0.01$, $p \leq 0.001$, respectively.

	MDR				MBO				H	V	H x V
	C	V2	V4	*Mean*	C	V2	V4	*Mean*			
Pre-planting phase											
Soluble proteins	36.49 a	32.6 a	35.78 a	*34.96*	42.33 a	33.45 a	41.56 a	*39.11*	ns	ns	ns
Ala	4.29 a	20.9 a	38.87 b	*21.35*	8.73 b	29.39 c	43.99 c	*27.37*	ns	***	**
Asn	59.72 a	65.39 b	156.8 a	*83.97*	32.24 a	50.37 c	88.12 d	*56.91*	ns	***	**
Asp	2.34	1.64	5.72	*3.23*	2.39	0.63	2.22	*1.75*	ns	**	*
GABA	2.42 a	16.28 b	32.59 c	*17.10*	6.63 d	29.72 d	65.57 d	*33.97*	ns	***	***
Gln	67.21 ab	39.05 b	105.8 c	*70.69*	51.56 ab	60.59 d	129.5 e	*80.55*	ns	***	***
Glu	20.92 a	31.17 a	54.86 a	*35.65*	23.95 b	42.79 c	46.53 bc	*37.76*	ns	***	*
Gly	2.18 ac	3.47 a	7.31 b	*4.32*	1.6 b	4.8 c	11.05 d	*5.82*	ns	***	***
MEA	3.43 a	3.07 a	6.55 a	*4.35*	2.62 b	5.76 bc	8.55 b	*5.64*	ns	***	***
Orn	0.9 a	1.18 a	2.32 ab	*1.47*	0.96 ab	1.67 b	2.8 b	*1.81*	ns	***	***
Pro	5.59 ac	9.97 a	11.36 b	*8.97*	6.77 a	6.83 b	13.84 c	*9.15*	ns	***	***
Ser	5.03 a	7.23 ab	13.63 ab	*8.63*	7.44 b	14.13 bc	19.43 c	*13.67*	*	***	***
Thr	8.92 a	7.13 b	20.44 b	*12.16*	6.77 a	9.00 a	19.46 a	*11.74*	ns	***	***

Total AA	228.5 ab	258.2 b	564 ab	*350.20*	196.4 ab	324.8 c	598.6 c	*373.30*	ns	***	***
BCAAs	17.68 ae	22.88 ae	53.25 a	*31.27*	18.19 ab	30.74 b	71.18 c	*40.04*	ns	***	**
Minor AA	45.61 a	51.75 a	107.7 b	*68.35*	44.73 c	69.14 c	147.5 d	*87.12*	ns	*	***
Vegetative phase											
Soluble Proteins	36.27 a	38.44 a	53.03 b	*45.58*	20.11 b	39.21 a	23.15 ab	*27.49 B*	*	ns	***
Ala	4.81 a	5.77 a	1.53 a	*4.04*	4.20 d	2.20 d	3.00 d	*3.13*	ns	*	***
Asn	687.1 e	175.2 f	157.8 cf	*340.00*	221 f	208.8 c	69.41 ad	*166.40*	ns	**	***
Asp	5.34	7.57	5.96	*6.29*	5.99	3.28	4.95	*4.74*	ns	ns	*
GABA	5.53 e	1.81 a	0.64 a	*2.66*	2.83 a	1.86 f	2.62 a	*2.44*	ns	*	***
Gln	87.61 ad	67.04 a	34.04 ab	*62.90*	64.00 b	56.4 cf	26.26 f	*48.89*	ns	**	***
Glu	48.11 c	18.13 ad	31.23 d	*32.49*	24.89 d	14.95 a	29.69 a	*23.18*	*	*	***
Gly	1.25 ae	1.2 a	0.54 a	*1 B*	2.03 a	1.95 e	1.14 a	*1.71 A*	**	*	***
MEA	16.12 c	15.9 c	7.01 c	*13.01*	15.67 b	5.93 b	4.58 b	*8.73*	*	*	***
Orn	2.9 b	2.85 b	2.01 b	*2.59*	3.37 bc	1.81 c	1.65 bc	*2.28*	ns	**	***
Pro	5.97 a	5.84 d	2.53 ad	*4.78*	3.35 d	3.44 d	6.04 a	*4.28*	ns	ns	***
Ser	4.83 a	2.63 ab	0.93 a	*2.80*	5.74 a	2.21 d	2.67 a	*3.54*	ns	***	*
Thr	12.17 a	2.12 ab	8.64 c	*7.64*	9.71 c	1.66 a	1.8 c	*4.39*	ns	**	***
Total AA	922.2 c	325.4 a	267.7 ab	*505.10*	410.6 a	314.5 ab	158.9 b	*294.70*	ns	**	***
BCAAs	11.63 d	6.93 e	4.16 f	*7.57*	13.04 f	4.3 f	6.25 f	*7.86*	ns	***	***
Minor AA	40.43 a	19.35 a	14.77 a	*24.85*	47.77 e	10.01 a	15.12 a	*24.30*	ns	***	***
Flowering phase											
Soluble Proteins	5.85 c	19.04 b	31.94 a	*18.94*	29.51 a	20.97 ab	14.68 ab	*21.72*	ns	ns	***

Ala	6.91 a	3.42 a	5.06 d	*5.13*	4.04 a	12.01 a	0.51 e	*5.52*	ns	ns	***
Asn	108.30 ac	80.17 a	120.40 a	*103.00*	76.37 ab	56.57 cd	0.02 g	*44.32*	**	ns	***
Asp	6.09	3.64	5.27	*5.00*	5.22	6.33	0.08	*3.88*	ns	ns	***
GABA	8.76 b	2.31 a	3.72 a	*4.93*	3.93 c	12.36 a	0.26 g	*5.52*	ns	ns	***
Gln	38.63 cf	36.47 ab	37.23 c	*37.44*	81.61 ad	79.08 cf	0.07 g	*53.59*	ns	*	***
Glu	44.66 ab	17.2 d	17.79 d	*26.55*	29.58 a	20.12 ad	2.86 f	*17.52*	ns	***	***
Gly	1.79 a	1.06 a	2.44 a	*1.76*	1.94 a	0.04	0.34 f	*1.09*	ns	ns	***
MEA	7.66 b	3.22 a	0.25 a	*5.63*	2.97 ab	3.36 b	2.27 a	*2.87*	**	ns	*
Orn	1.06 a	1.02 a	0.54 a	*0.87*	0.87 c	1.81 a	0.44 a	*1.04*	ns	**	***
Pro	6.66 a	3.63 ad	12.07 ad	*7.45*	3.95 b	11.38 bc	2.2 d	*5.84*	ns	ns	***
Ser	3.44 a	3.1 a	3.74 a	*3.43*	3.45 a	3.91 a	0.14 e	*2.50*	ns	ns	***
Thr	2.83 bc	2.81 bc	3.97 bc	*3.20*	2.91 b	4.4 b	0.08 d	*2.46*	ns	ns	***
Total AA	256 ab	173.9 ab	231.1 b	*220.30*	233.5 ab	231.6 ab	12.03 d	*159.00*	ns	ns	*
BCAAs	8.22 f	6.02 f	7.69 f	*7.31*	5.75 f	6.83 f	1.27 g	*4.62*	*	ns	***
Minor AA	19.18 a	15.82 a	12.9 a	*15.97*	16.72 ae	19.27 a	2 e	*12.66*	ns	**	***

Supplemental file S1

Chromatogram of the standard amino acids mixture (40 µM) in full scale (A) and zoom window (B)

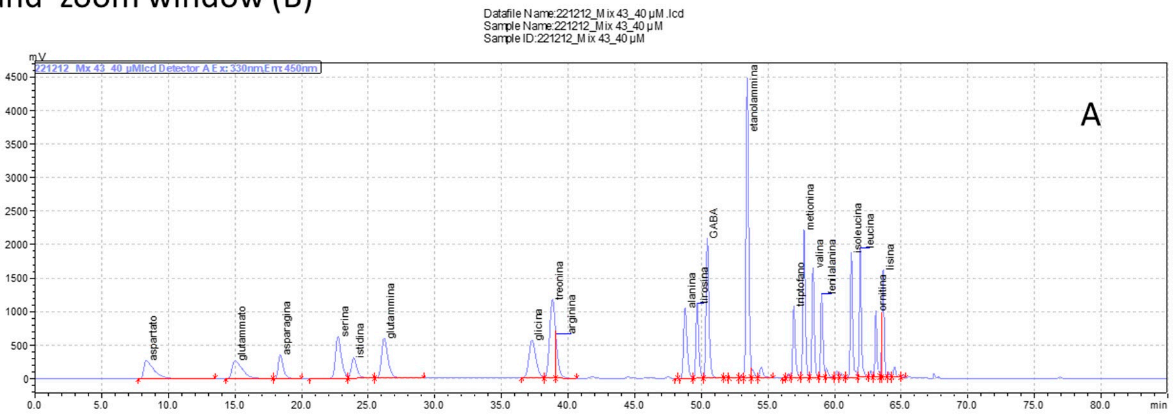

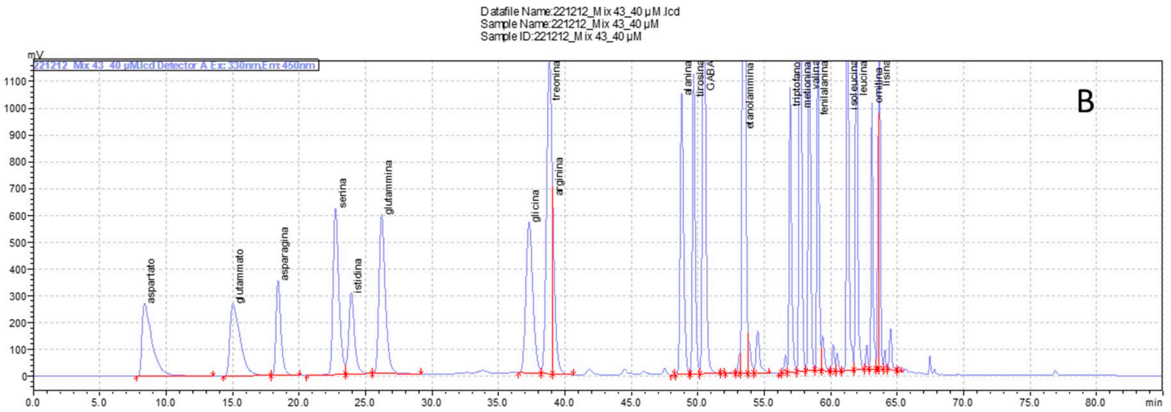

Chromatogram of the amino acids profile of one of the three biological replicates for MDR hybrid in pre-planting phase in full scale (C) and zoom window (D)

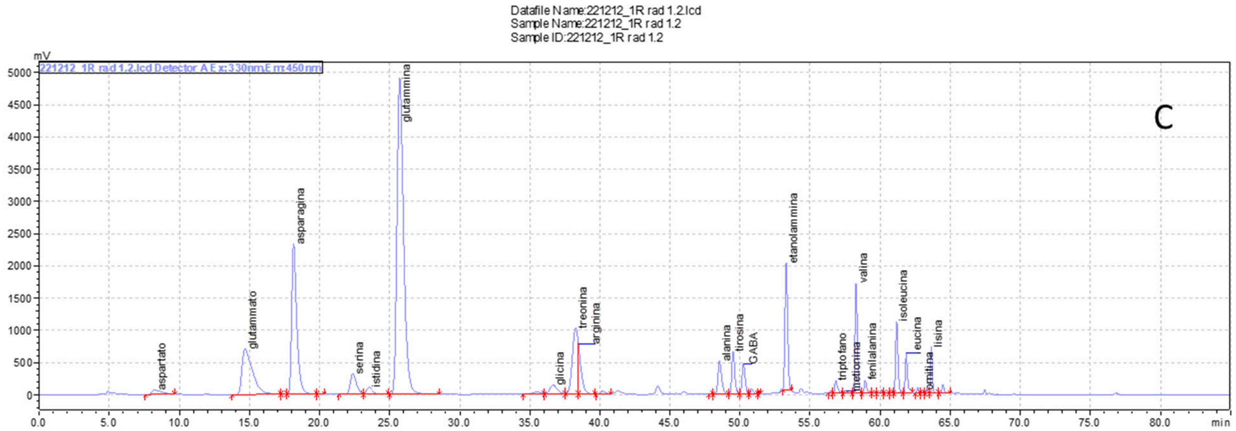

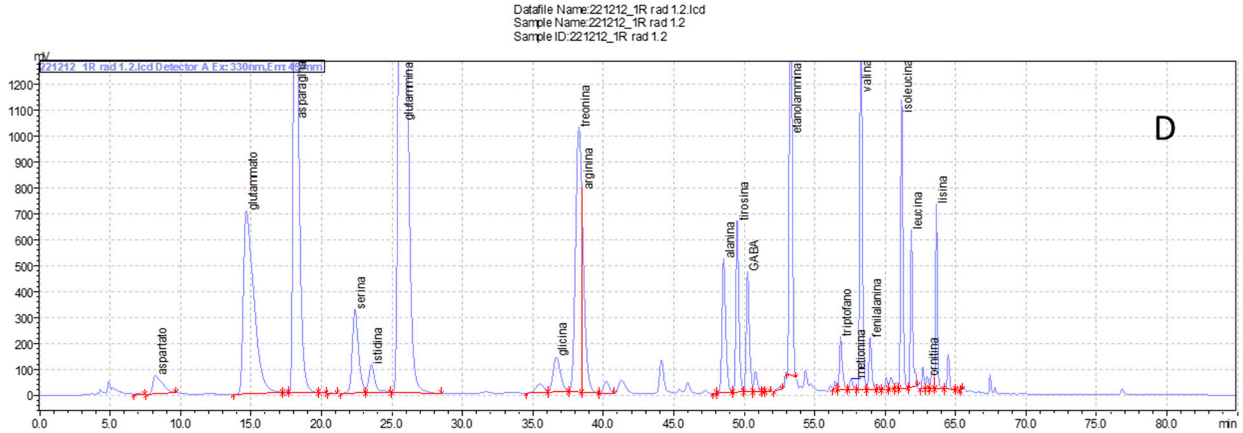

Chromatogram of the amino acids profile of one of the three biological replicates for MBO hybrid in pre-planting phase in full scale (E) and zoom window (F)

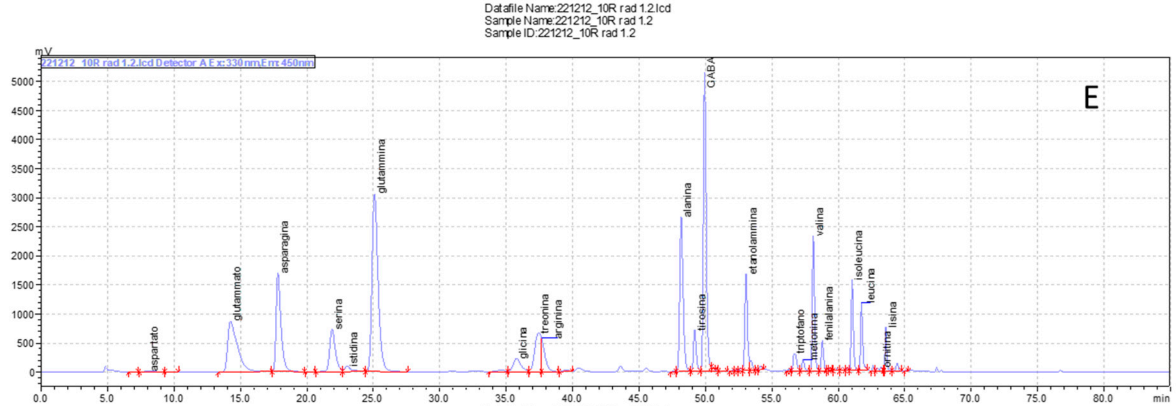

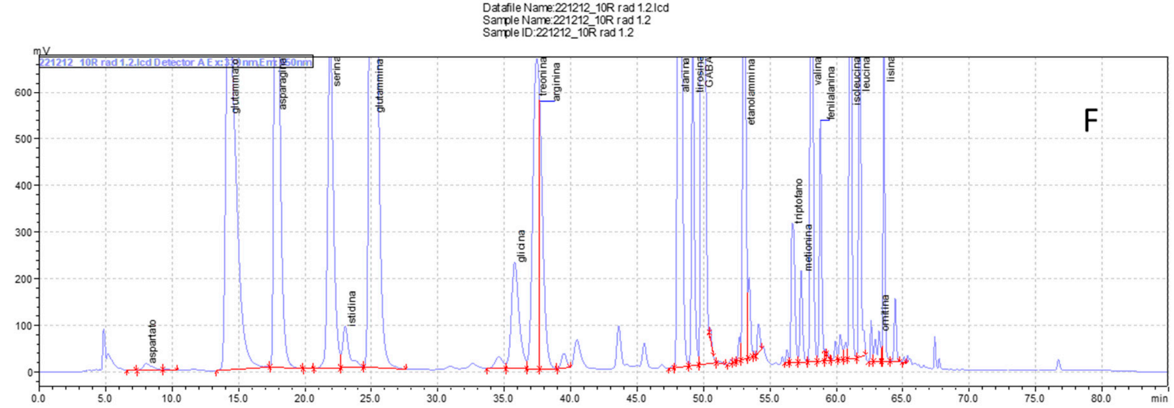

Supplement: Supplementary file 1 [file plants-12-03255-s001.zip › plants-2574778-supplementary.pdf]
